# Integrating AI-Powered Chatbots Into Patient Education From the Perspectives of Patients, Caregivers, and Nurses: Qualitative Study

**DOI:** 10.2196/87254

**Published:** 2026-07-30

**Authors:** Zihao Liu, Yuli Li, Jingjing Wang, Qing Liu, Feifei Chen, Lifeng Zhu, Yanbei Ren, Linlin Xing, Xiaoyun Wang

**Affiliations:** 1 School of Nursing and Rehabilitation Cheeloo College of Medicine Shandong University Jinan, Shandong China; 2 Department of Nursing The Second Qilu Hospital of Shandong University Jinan, Shandong China; 3 Department of Cardiology Qilu Hospital of Shandong University Jinan, Shandong China; 4 Department of Neurology Caoxian People's Hospital Heze, Shandong China

**Keywords:** AI-powered chatbot, patient education, nurse-patient trust, nursing, digital health literacy

## Abstract

**Background:**

AI-powered chatbots offer new opportunities to enhance patient education; however, their integration may reshape patterns of information interactions and trust relationships among patients, caregivers, and nurses. Evidence remains limited on how these stakeholders perceive the value and risks of AI-powered chatbots, and on their potential effects on nurse-patient trust.

**Objective:**

This study explores patients’, caregivers’, and nurses’ attitudes toward and experiences with integrating AI-powered chatbots into patient education and identifies perceived benefits, implementation challenges, potential effects on trust, and the supportive conditions required for safe integration.

**Methods:**

This qualitative study was conducted from April to July 2025. Patients and caregivers were recruited from a tertiary general hospital using maximum variation purposive sampling, while nurses were recruited through snowball sampling from 6 hospitals of varying tiers. Data were collected using a sociodemographic questionnaire and semistructured, in-depth interviews. Interview recordings were transcribed verbatim and analyzed using reflexive thematic analysis, with NVivo used for coding and theme development. Sociodemographic data were analyzed descriptively.

**Results:**

A total of 60 participants were included: 29 patients, 17 caregivers, and 14 nurses. Four themes were identified: perceptions and maintenance of nurse-patient trust, conditional acceptance and practical needs, functional optimization and implementation safeguards, and nurses’ role pressures and competency restructuring. All 3 stakeholder groups recognized the potential of AI-powered chatbots to address unmet information support needs in patient education but expressed reservations about their accuracy, personalization, and transparency. AI-powered chatbots were not perceived as a direct threat to nurse-patient trust. However, nurses were more sensitive to potential trust tensions, increased explanation burden, and expanded professional responsibilities, highlighting the need for competency restructuring. Stakeholder groups also differed in their perceptions of the conditions required to maintain nurse-patient trust. Limited digital health literacy and the digital divide affecting older patients were major barriers to integrating AI-powered chatbots into patient education.

**Conclusions:**

Patients, caregivers, and nurses were generally cautiously open to integrating AI-powered chatbots into patient education, although their assessments of benefits and risks differed by role. AI-powered chatbots may be best positioned as adjunctive information-support tools. Their safe use should be tailored to patient characteristics, information risk, and clinical context, with nurses’ professional oversight and coordinated support across governance, technical, and clinical implementation levels.

## Introduction

Patient education is a core component of patient-centered care and extends across the continuum of treatment, rehabilitation, and long-term disease management [1]. It plays an important role in improving patients’ understanding of their condition [2], enhancing treatment adherence, and supporting self-management [3-6], with nurses serving a central role in its delivery [7]. However, high patient-to-nurse ratios, limited time for communication, and heavy clinical workloads often constrain the provision of comprehensive patient education. As a result, patients’ disease-related information needs may remain unmet [8,9], potentially compromising their self-management capacity and health outcomes [10].

Against this background, AI-powered chatbots offer a new approach to supplementing existing patient education through natural language interaction, immediate responses, and continuous availability. Previous studies have explored their use in answering patients’ health-related questions, explaining complex medical information, and supporting patient-clinician communication, with preliminary evidence suggesting that they may improve the accessibility and readability of health information and enhance patient understanding [11,12]. Generative AI may also assist health care professionals with clinical documentation and the development and simplification of patient education materials, thereby helping reduce workload and improve the quality of care [13-15]. Overall, AI-powered chatbots may expand the channels through which patients access health information and provide adjunctive support to health care professionals in delivering patient education, thereby offering new opportunities to improve current patient education practices.

Notably, the integration of AI-powered chatbots into patient education may reshape existing information interactions and relational structures. Previous research suggests that information interactions, traditionally occurring primarily between patients and health care professionals, may evolve into a triadic relationship involving patients, professionals, and AI-powered chatbots [16]. This shift may have both beneficial and adverse effects [16]. Immediate and interactive information support may enable patients to participate more actively in clinical communication. However, biased information, limited transparency in content generation, and inconsistencies with professional advice may undermine trust and interpersonal relationships between patients and health care professionals [17,18]. Effective patient education depends on sustained communication, shared participation, and a strong therapeutic relationship between patients and health care professionals [19]. As key providers of patient education, nurses rely on nurse-patient trust to maintain open communication, promote collaboration in care, and support the effectiveness of patient education. Therefore, whether and how AI-powered chatbots reshape existing information interactions and nurse-patient trust in patient education warrants close attention. However, research on AI-mediated relationships between patients and health care professionals has focused mainly on physician-patient interactions [20,21]. Evidence remains limited regarding potential changes in nurse-patient trust within nurse-led patient education.

Beyond its potential effects on information interactions and nurse-patient trust, the safe and acceptable integration of AI-powered chatbots into patient education also depends on how different stakeholders perceive their value, potential risks, and appropriate boundaries for use. Although previous studies have extensively examined AI-powered chatbots and large language models across a range of patient education tasks, most have focused on technical performance, including the accuracy, completeness, and readability of generated content [22,23]. Such evaluations provide limited insight into how these tools are used and accepted in real-world patient education settings. Qualitative evidence suggests that chatbot adoption depends not only on usability and technical performance but also on trust, privacy, cultural appropriateness, and the involvement of health care professionals [24]. However, within nurse-led patient education, empirical evidence remains limited regarding how different stakeholders perceive the benefits and risks of AI-powered chatbots and how their use may affect trust relationships. Caregivers also play an important role in patients’ daily care and health-related decision-making, yet their experiences have received comparatively little attention [25,26].

Accordingly, this study explored patients’, caregivers’, and nurses’ attitudes toward and experiences with integrating AI-powered chatbots into patient education. It examined their potential effects on information interactions and nurse-patient trust and identified the perceived benefits, challenges, and supportive conditions required for safe integration, thereby providing empirical evidence to inform tool development and clinical implementation.

## Methods

### Study Design

This study used a qualitative descriptive design [27] to explore patients’, caregivers’, and nurses’ attitudes toward and experiences with integrating AI-powered chatbots into patient education, as well as their implementation needs. This design enabled the researchers to remain close to the data and provide a clear, detailed account of participants’ perspectives and experiences within their real-world contexts [28-30]. The study was informed by a pragmatic orientation, emphasizing the understanding of practical clinical problems and the implications of the findings for the safe integration of AI-powered chatbots into patient education [31].

Qualitative data were analyzed using reflexive thematic analysis [32], a theoretically flexible approach for identifying, interpreting, and reporting patterns of meaning within participants’ accounts. Researchers maintained reflexivity throughout the analysis by considering how their positions influenced data generation and interpretation [32,33]. Reporting followed the COREQ (Consolidated Criteria for Reporting Qualitative Research) checklist [34] (see Multimedia Appendix 1).

### Participants and Recruitment

Participants were recruited and interviewed between April and July 2025.

### Patients and Caregivers

Patients and caregivers were recruited in person from multiple wards of a large tertiary general hospital in Jinan, Shandong Province. Patients were eligible if they were aged 18 years or older, clinically stable, able to communicate, and had experience receiving patient education. Those who were unable to complete the interview because of physical discomfort, cognitive impairment, severe mental health problems, or communication difficulties were excluded. Caregivers were eligible if they were aged 18 years or older, served as the patient’s primary informal caregiver, participated in daily care and health-related decision-making, and were able to communicate. Individuals with cognitive or communication impairments and paid caregivers were excluded.

Potential participants were identified and assessed for eligibility through a 2-stage process. The researchers first reviewed demographic and clinical information in the medical records, including age, educational attainment, and disease status, and consulted the nurse responsible for each patient to identify patients who were likely to meet the eligibility criteria and had experience receiving patient education. Ward nurses also helped identify potential primary informal caregivers. Clinical staff were involved only in the initial identification and contact, whereas final eligibility was determined by the research team. During the patient’s hospitalization, the responsible nurse facilitated initial contact between the researchers and potential participants. The researchers then approached potential participants face to face in the ward, explained the study purpose and procedures, answered questions, and obtained informed consent. Any eligibility information that remained uncertain was subsequently verified. For caregivers, the researchers confirmed that they were the patient’s primary informal caregiver, participated in daily care and health-related decision-making, and provided care without payment. Final eligibility was determined based on this information. Patients and caregivers were also asked about their previous use of AI-powered chatbots to inform subsequent maximum variation sampling. Only participants who met all eligibility criteria completed the sociodemographic questionnaire and interview.

Participants were recruited using maximum variation purposive sampling [35]. Selection focused on characteristics such as age, educational attainment, and prior experience with AI-powered chatbots, as these factors may influence individuals’ ability, willingness, and acceptance of digital technologies [36,37] and were closely aligned with the research questions. Recruitment decisions were made dynamically by the research team according to the inclusion criteria and the evolving composition of the sample. Individuals who increased sample diversity and provided information-rich perspectives were prioritized.

### Nurses

Eligible nurses had at least 1 year of clinical experience to ensure sufficient familiarity with the clinical setting. Nurses were recruited using snowball sampling [38]. The research team first identified initial nurse participants through professional networks and then asked them to recommend other nurses who met the inclusion criteria and could provide relevant insights. During referral and screening, the research team considered nurses’ years of clinical experience, specialty background, patient education experience, and experience using AI-powered chatbots when selecting subsequent participants to capture diverse nursing perspectives.

### Data Collection

The research team developed the interview guide (Multimedia Appendix 2) through discussion, informed by the literature review and the study objectives. Trained interviewers conducted the interviews using this guide. Before formal data collection, pilot interviews were conducted with 5 patients or caregivers and 5 nurses to assess the clarity, relevance, and appropriateness of the questions. The guide was refined based on participant feedback and team discussion. As only minor revisions were made, the pilot interviews were included in the final analysis.

Interviews with patients and caregivers were conducted primarily face-to-face at the bedside. At times, a caregiver and up to 2 other patients in the room were present. Privacy curtains were used, and interviews were scheduled during quieter periods in the most private space available within the ward to create a semiprivate environment. To minimize potential influence among participants sharing the same room, only 1 participant was interviewed from each room. Interviews explored participants’ experiences with AI-powered chatbots, attitudes toward their integration into patient education, perceived influencing factors, and functional and support needs. Interviews with nurses were conducted primarily online via Tencent Meeting (Tencent Holdings Ltd), with no nonparticipants present. These interviews explored the effects of AI-powered chatbots on current patient education, nurse-patient interactions, and trust relationships, as well as implementation challenges and competency development needs.

Before each interview, the interviewer introduced themselves and explained the study purpose and interview topics. Participants were informed that their responses would not affect the medical or nursing care they received. Confidentiality and voluntary participation were emphasized, including participants’ right to decline to answer any question or withdraw from the study at any time. With participants’ consent, interviews were audio-recorded and transcribed verbatim within 24 hours. Interviews lasted 10-48 minutes. All transcripts were deidentified and assigned unique codes.

Data collection and preliminary analysis proceeded concurrently. Interviews with patients, caregivers, and nurses were coded and analyzed separately before themes were developed across the data. The research team continually assessed the richness, diversity, relevance, and explanatory value of the data in relation to the study objectives. Sample size was not determined solely by the absence of new codes or themes. Instead, following the guidance of Braun and Clarke [39], decisions about when to stop data collection were informed by the concept of information power, which emphasizes the richness, relevance, and quality of interview data [40]. Data collection ended after 60 interviews, when the research team determined that the information power was sufficient to address the study objectives.

In addition to the qualitative data, after obtaining participants’ consent and before the formal interview, the researchers used a brief structured sociodemographic questionnaire to collect participants’ general characteristics. All information was self-reported.

### Data Analysis

Participant characteristics were summarized using descriptive statistics, and categorical variables were presented as counts and percentages. AI-powered chatbot use was cross-tabulated by variables including age, gender, and educational level. Data were analyzed using IBM SPSS Statistics (version 27.0) and Microsoft Excel.

Qualitative data were analyzed using reflexive thematic analysis based on Braun and Clarke’s [41] 6-phase framework, with NVivo software (version 15.0; Lumivero) used to support the analysis. The analysis was primarily inductive. Initial coding was not guided by a predefined theoretical framework or a fixed coding manual, although the study objectives provided an overall focus for data familiarization and analysis. Initial codes remained close to participants’ explicitly expressed views and experiences. During theme development, the research team compared accounts across participant groups and specific contexts of use to interpret the relationships, differences, and contextual meanings underlying these perspectives.

The analytic process was recursive and iterative [32]. ZL conducted the primary coding of the full data set, while YL reviewed coded extracts, analytic memos, and candidate themes and engaged in ongoing reflexive discussions with ZL. Other team members contributed by reviewing transcripts and candidate themes, challenging preliminary interpretations, and considering alternative explanations. Patient, caregiver, and nurse data were initially analyzed separately and then compared across groups to identify commonalities, differences, and tensions among stakeholder accounts.

Candidate themes were developed and refined through repeated review of coded extracts and the full data set. The research team held regular reflexive discussions to assess whether the themes adequately represented participants’ accounts, with attention to negative cases, discrepant views, and alternative interpretations. Differences in team members’ interpretations were treated as resources for deepening the analysis rather than as coding errors to be resolved through consensus [32,42]. Finally, the team refined the theme names and definitions and selected quotations to illustrate the analytic claims. A detailed coding tree showing the themes, subthemes, and codes is presented in Table S4 in Multimedia Appendix 3.

### Researcher Characteristics, Reflexivity, and Rigor

Interviews were conducted primarily by 2 female researchers, ZL and YL, who had received training in qualitative interviewing. ZL was a master’s student, and YL was a faculty member with a doctoral degree and experience in qualitative research. Neither interviewer was involved in the participants’ diagnosis, treatment, or nursing care, and neither had a prior clinical relationship with them.

The research team recognized that the researchers’ professional backgrounds and academic interest in AI-assisted health care, as well as power dynamics and limited privacy during bedside interviews, could influence data generation and interpretation. These potential influences were continually examined through reflexive documentation and regular team discussions. Rigor was supported through sustained engagement with the data, repeated review of the themes against the full data set, and attention to negative cases, divergent views, and differences across participant groups. Illustrative quotations were used to support the analytic interpretations. Further details on researcher reflexivity, strategies to ensure rigor, and data analysis are provided in Multimedia Appendix 3.

### Ethical Considerations

This study was approved by the Ethics Committee of the School of Nursing and Rehabilitation, Shandong University (approval number 2025-R-077). All participants provided written informed consent. Deidentified data were used to protect participants’ privacy. Participants did not receive any compensation. All procedures adhered to the ethical principles of participant autonomy and safety.

## Results

### Participant Characteristics and AI-Powered Chatbot Use

This study included 60 participants: 29 patients, 17 caregivers, and 14 nurses. During recruitment, 2 patients and 3 caregivers declined to participate, mainly because of limited interest or time. The mean ages of patients and caregivers were 37.9 (SD 12.73) years and 37.12 (SD 11.79) years, respectively. Among them, 22 patients (76%) and 12 caregivers (71%) had used AI-powered chatbots, most frequently to seek treatment-related information. The nurses had a mean age of 34.64 (SD 5.85) years and a mean of 10.1 (SD 5.3) years of clinical experience. All had used AI-powered chatbots in clinical practice, but only 4 (29%) had used them for patient education. Additional participant characteristics are presented in Tables S1-S3 in Multimedia Appendix 3. AI-powered chatbot use also showed a clear gradient across educational levels (Figure S1 in Multimedia Appendix 3).

**Figure 1:**
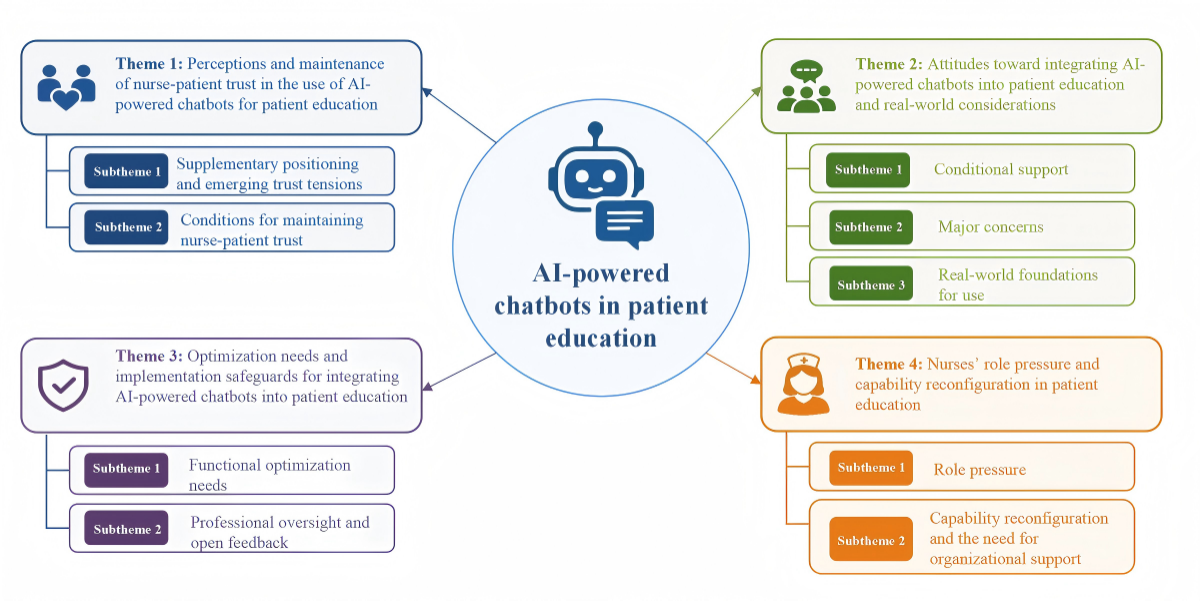
Overview of themes and subthemes related to integrating AI-powered chatbots into patient education.

Reflexive thematic analysis identified 4 themes and 9 subthemes, which are summarized in Figure 1. Although several perspectives were shared across participant groups, patients, caregivers, and nurses also differed in their concerns and role-specific emphases. A detailed cross-group comparison of these commonalities and differences is provided in Table S5 in Multimedia Appendix 3. In the participant quotations, P, C, and N denote patients, caregivers, and nurses, respectively.

### Theme 1: Perceptions and Maintenance of Nurse-Patient Trust in the Use of AI-Powered Chatbots for Patient Education

#### Overview

AI-powered chatbots were not viewed as a direct threat to nurse-patient trust because they were seen as supplementary tools rather than replacements for nurses’ core professional role. However, participants differed in their perceptions of potential threats to trust and the conditions needed to maintain it.

#### Subtheme 1: Supplementary Positioning and Emerging Trust Tensions

AI-powered chatbots were mainly viewed as supplementary tools for patient education. At present, participants did not perceive their use as clearly undermining nurse-patient trust. Patients and caregivers used them primarily to supplement information and improve their understanding rather than for independent diagnosis.

When I have questions and want to learn more about my condition, I use it. It helps me understand some information in advance, so I can better cooperate with the doctors and have a clearer understanding of my illness.

P13

Nurses primarily used AI-powered chatbots for supportive tasks, such as editing written materials and preparing nursing documentation. They had not yet incorporated them directly into nurse-patient interactions during patient education.

Patients and caregivers also viewed chatbot-generated information as insufficiently personalized and continued to place greater trust in health care professionals. This further reduced the perceived impact of AI-powered chatbots on nurse-patient trust.

The nurse still explains things more comprehensively and in greater detail. What you find through the chatbot is usually quite general...Sometimes I just use it for comparison, but the nurse is still more accurate.

C6

A few nurses reported that patients or caregivers questioned the educational information they provided after consulting AI-powered chatbots. Although nurses were able to clarify these discrepancies, such encounters made them feel challenged and increased their communication and explanatory burden.

At that time, he raised this concern, and we gave him a very clear explanation. But even after we explained it, he was still only half convinced...If it was something we genuinely could not explain, I would feel a little uneasy.

N8

Other nurses had not personally encountered similar situations but expressed similar concerns.

When there are inconsistent answers, I would feel questioned, and it would make communication with the patient much more challenging.

N4

Overall, AI-powered chatbots were not currently perceived as a direct threat to nurse-patient trust. However, when chatbot-generated information conflicts with professional explanations and prompts patients to question the information provided by nurses, chatbots may shift from being a source of informational support to a potential source of tension in nurse-patient communication.

#### Subtheme 2: Conditions for Maintaining Nurse-Patient Trust

Although AI-powered chatbots were not currently viewed as a direct threat to nurse-patient trust, participants identified several conditions needed to preserve trust as their use in patient education expands.

Some patients and caregivers emphasized that nurses should remain visibly involved. AI-powered chatbots could assist with patient education but should not replace nurses’ explanations, guidance, and oversight.

If it is introduced into clinical practice, that is fine, but it should not affect (the quality of treatment). For example, if all the tasks are assigned to AI and nurses no longer do what they should do, that would not be good. Nurses can work together with AI, and AI can serve as an auxiliary tool.

P23

This suggests that nurses’ visible professional involvement is essential to maintaining nurse-patient trust when AI-powered chatbots are used to support patient education.

Some participants also viewed emotional support as important. Nurses’ care, empathy, and responsiveness during face-to-face interactions were considered irreplaceable.

Care, especially emotional care, is something artificial intelligence simply cannot replace.

P6

However, a few patients and caregivers offered a more nuanced perspective. Although AI-powered chatbots cannot provide genuine empathy, their immediate and continuous responses could offer reassurance. This was especially helpful before seeking care, when professional support was unavailable, or when users hesitated to contact health care professionals repeatedly. In these situations, chatbots could support initial understanding and reduce anxiety and relationship strain arising from uncertainty and repeated questions.

Sometimes, he (the health care professional) may also be in a bad mood. But if you use an AI chatbot, there is no such thing as being in a bad mood. Whatever you ask, it will definitely respond to you. At the very least, it provides some emotional value.

P17

Nurses further emphasized that maintaining trust required strong professional competence, particularly sound clinical knowledge. Patients and caregivers likewise trusted nurses who provided accurate, professional explanations, highlighting professional competence as a key foundation of nurse-patient trust.

If you are familiar with the whole work process and with disease-related nursing care, then when patients ask questions, you can answer them accurately, and patients will trust you.

N11

In addition, participants also noted that AI system maturity, patients’ cognitive ability, and the quality of nurse-patient communication could shape the impact on trust. When patients struggled to understand AI-generated content, additional information sources could increase misunderstanding and conflict.

It depends on (patients’) personal cognition. Some patients may not fully understand these issues, which could increase the possibility of conflict.

P26

This also highlights the importance of nurses’ contextual communication skills in maintaining nurse-patient trust. Providing individualized communication tailored to patients’ specific circumstances may help prevent conflicts.

Some people are easy to communicate with. When you explain things to them, they are happy. But some people directly question you, and this can create a sense of distrust. In such cases, I pay more attention when communicating with them.

N4

### Theme 2: Attitudes Toward Integrating AI-Powered Chatbots Into Patient Education and Real-World Considerations

#### Overview

This theme shows that acceptance was conditional rather than a general endorsement of AI. Participants supported AI-powered chatbots when they could address information gaps in specific patient education contexts without introducing new risks related to technical limitations or differences in users’ abilities.

#### Subtheme 1: Conditional Support

Participants generally supported integrating AI-powered chatbots into patient education, but only under certain conditions. They valued their potential to provide convenient, comprehensive information support. However, concerns about accuracy, personalization, transparency, and clinical relevance limited their acceptance of direct use. Further improvements were therefore considered essential for broader acceptance and implementation.

I am willing to have it introduced, but at this stage, I think it may not be very appropriate to introduce it into clinical practice. It still feels not advanced enough and needs further improvement, especially in improving the accuracy of the information it provides.

P8

Patients and caregivers without prior experience using AI-powered chatbots relied more on health care professionals and remained cautious about chatbot-generated information.

Nurses’ attitudes were more context-dependent than those of patients and caregivers. They emphasized that health care professionals should remain the primary source of information when professional support is available, and the information requires substantial clinical expertise. In out-of-hospital settings or when continuity of support is limited, AI-powered chatbots could provide supplementary information. Nurses also considered chatbots more suitable for low-risk, broadly applicable topics, such as diet and physical activity, than for high-risk information related to clinical decision-making.

I think when patients are hospitalized and health care professionals are right there, they should communicate with them directly if they have questions. After all, the attending physician will consider the patient’ s own condition and test results. Patients do not really understand their actual situation, because they do not have a medical background, and their searches are certainly not targeted...For discharged patients, when continuity of care for most diseases is not well provided, especially when it is a relatively simple question, patients can be asked to look it up themselves.

N14

Overall, participants’ acceptance of AI-powered chatbots was not simply a matter of support or opposition but rather a conditional acceptance based on technological maturity, the level of information risk, and the specific context of use.

#### Subtheme 2: Major Concerns

Beyond technical maturity, participants were also concerned about whether AI-generated information could be readily understood and used effectively.

Nurses viewed limited digital health literacy among patients and caregivers as a key concern. When users lacked medical knowledge or were unable to ask appropriate questions or evaluate the information in the context of their own condition, AI-generated content could lead to information overload, misunderstanding, or greater illness uncertainty rather than improve understanding. This risk was considered greater for patients with complex conditions, such as cancer or multiple comorbidities.

Patients with malignant tumors are worried, so they use AI to search for information. But they do not understand many technical terms, and they do not know the specific situation of their own disease. They just search randomly, which instead increases their uncertainty about the disease.

N4

This concern was also reflected in the experiences of patients.

Because I do not understand it, I become worried when I look at the results shown.

P22

Nurses added that when patients and caregivers could not effectively assess AI-generated information, additional information sources could increase nurses’ explanatory burden and workload.

Some patients do not understand the information generated by AI-powered chatbots, and they also come to ask us about information that is only minimally related to their disease. This instead increases our workload.

N12

The suitability of AI-powered chatbots for older adults highlighted both the digital divide and the need for digital empowerment. Patients, caregivers, and nurses noted that older adults often struggled to use smart devices, ask questions, and understand chatbot responses. Caregivers and nurses suggested that traditional education or simpler formats, such as short videos, might be more suitable.

Some older adults cannot even use smartphones, so naturally they cannot use artificial intelligence. Older adults face many difficulties (in seeking medical care).

P23

At present, our department still has many older patients. They may use this (AI-powered chatbots) relatively little. Some traditional or short, fragmented materials (such as short videos) may be more suitable for them. Asking them to raise questions may not be very appropriate.

N5

However, older adults were not a homogeneous digitally disadvantaged group. Some expressed a willingness to learn and use AI-powered chatbots to strengthen their autonomy in seeking care and reduce their dependence on family caregivers.

If you do not learn, it is difficult even to seek medical care now. Everything requires scanning a code on a mobile phone. You cannot always rely on your children because they have their own family responsibilities, so you have to manage it yourself.

P9

These findings suggest that patient education approaches should be tailored to individuals’ digital capabilities, health literacy, and available caregiving support.

#### Subtheme 3: Real-World Foundations for Use

Gaps in conventional patient education across different care settings also created a practical basis for integrating AI-powered chatbots into patient education.

In primary care and remote areas, limited medical resources and specialist expertise made chatbots a supplementary source of disease-related information. A few patients trusted AI-generated information more than information provided by primary care institutions. This perspective suggests that chatbot use may reinforce perceived differences in professional credibility across levels of the health care system.

Compared with primary care hospitals, I trust AI. AI must collect authoritative opinions. Although doctors in county hospitals have medical ethics, they have encountered fewer cases and have less practice, so they are still somewhat lacking.

P17

In inpatient settings, nurses’ heavy workloads and limited time for patient education also supported the use of AI-powered chatbots as supplementary tools. Patients and caregivers found some disease and care information too technical to fully understand during brief nurse-patient interactions. They therefore used chatbots for further explanation and information after these encounters. Chatbots also supported nurse-patient communication. By explaining professional information and clarifying concepts, they helped patients and caregivers communicate with nurses more actively and with better preparation. Caregivers added that this support helped them participate in patient care and related decision-making.

Before we used AI, communication with health care professionals was one-way because we did not understand anything and simply accepted whatever the doctor said...Now, with AI, we can learn something in advance and communicate more effectively.

C11

Nurses also valued AI-powered chatbots for supplementing and explaining educational information. From their perspective, chatbots could reduce workload and improve efficiency by saving time on nursing documentation and simple, repetitive educational tasks. This could free nurses to focus more on individualized communication and complex concerns, thereby improving the quality of patient education.

In out-of-hospital settings, limited post-discharge follow-up and continuity of patient education created another practical need. Patients, caregivers, and nurses noted that existing services could not always provide timely and ongoing professional support at home, especially for chronic disease management. AI-powered chatbots were therefore viewed as a potential means of extending the continuity of patient education beyond discharge.

Health management is actually an ongoing issue for chronic diseases...I think it would be better to use AI as a follow-up system.

N13

### Theme 3: Optimization Needs and Implementation Safeguards for Integrating AI-Powered Chatbots Into Patient Education

#### Overview

This theme shows that safe integration requires more than technical improvement. Professional regulation, nurse oversight, feedback mechanisms, and organizational support are also needed to ensure the safe and appropriate use of AI-powered chatbots in patient education.

#### Subtheme 1: Functional Optimization Needs

Patients, caregivers, and nurses viewed greater accuracy and personalization as the most urgent improvements. They suggested adding more real-world clinical cases to strengthen the database and developing health care–specific AI chatbots overseen by government or professional bodies to improve credibility and clinical relevance.

There are too few cases. More cases should be fed to it (the AI-powered chatbot), such as participating in doctors’ consultations...Through this repeated training process, it will become more accurate and closer to human judgment.

P7

However, the use of real-world clinical data to improve system performance also raised concerns about privacy and data security.

whether people are willing to have their own data included

P10

These findings suggest that optimizing AI-powered chatbots should involve more than improving accuracy. Greater clinical relevance must be balanced with ethical data use and privacy protection.

Patients, caregivers, and nurses also stressed the importance of professional platforms and external oversight. They called for health care–specific chatbots regulated by government or professional bodies to address “who will regulate it and who will ensure its authenticity and professionalism” [C13]. From a clinical application perspective, nurses further highlighted the importance of developing nursing-specific education platforms.

With the help of large models, we can configure personalized, department specific educational content in the background, which will completely change today’ s cumbersome one-to-one education model.

N4

Patients and caregivers also emphasized accessibility and equity when integrating AI-powered chatbots into patient education. They recommended simpler operation, multiple input and output options, clearer responses, and no added financial burden. Participants warned that high technical or financial barriers could widen existing disparities in access to health information.

People already bear a huge burden of medical expenses. We cannot let the introduction of these tools make care more expensive to the point that ordinary people cannot afford it. This would also be unfavorable to the development of artificial intelligence.

P9

Given that the functions of AI-powered chatbots still require further improvement, patients and caregivers also suggested a gradual implementation pathway, moving from simple to more complex applications.

It should still be introduced in stages and batches. Use it for simple or certain tasks, while uncertain and complex tasks should still be handled by humans.

C10

Nursing managers further noted that systematic implementation would require appropriate infrastructure, including devices, patient access controls, system maintenance, and mechanisms for monitoring information quality.

You need to consider the equipment, appropriate patient access, safeguards for the accuracy of retrieved information, and a system maintenance plan.

N8

#### Subtheme 2: Professional Oversight and Open Feedback

Given the complexity of medical information and patients’ and caregivers’ limited medical knowledge, participants believed that chatbot use should not rely on users searching for and interpreting information independently. Nurse oversight and guidance were considered essential.

Patients and caregivers wanted nurses to help improve their search strategies, verify information accuracy, and assess whether AI-generated content was appropriate for the patient’s condition. This could reduce misunderstanding and misuse. Nurses likewise emphasized the need for professional guidance and review.

(Nurses) should tell me whether the information I found is correct, or how to search more precisely.

P13

I would search first to make sure the terms are correct, and then guide patients to ask questions in the right direction.

N1

Patients and caregivers expected support from nurses but were reluctant to disclose their chatbot use. They feared stigma or tension with nurses. This suggests that feedback mechanisms for discussing AI-generated information in patient education remain limited.

When talking with health care professionals, I pay special attention to this. I cannot say, “I saw this online.” As health care professionals, they may not want to hear this, so I try to avoid mentioning it.

P9

This shows that AI-powered chatbots do not reduce nurses’ central role in patient education. Instead, they further highlight nurses’ essential roles in guiding use, interpreting information, initiating communication, and ensuring information quality.

### Theme 4: Nurses’ Role Pressure and Capability Reconfiguration in Patient Education

#### Overview

The introduction of AI-powered chatbots expanded the boundaries of nurses’ responsibilities in patient education while also creating pressure related to knowledge updating and perceived professional competence. Adapting to these role changes required not only the development of individual capabilities but also support at the organizational and policy levels.

#### Subtheme 1: Role Pressure

The use of AI-powered chatbots in patient education introduced new pressures for nurses. Nurses noted that as patients and caregivers gained access to a wider range of information sources, they faced greater pressure to keep their professional knowledge up to date. When patients raised questions beyond nurses’ existing knowledge, this could undermine their sense of professional competence. Such concerns appeared particularly pronounced among nurses with fewer years of clinical experience. A nurse with 1.5 years of clinical experience noted:

I may not be able to fully answer patients’ questions, because I have tried using chatbots myself and feel that the content they provide is more comprehensive than my current understanding. I would feel worried.

N11

#### Subtheme 2: Capability Reconfiguration and the Need for Organizational Support

To address these pressures, nurses identified several areas for capability development. Continual updating of professional knowledge and improved competence in using AI tools were viewed as foundational requirements.

We need to enrich our professional knowledge, and we also need to learn how to use AI. We need to understand AI and learn how to use it.

N8

Nurses also viewed critical thinking and evidence-based practice as essential for evaluating AI-generated information and ensuring safe patient education. As chatbots may change what and how patients ask questions, nurses also need stronger contextual communication and adaptive response skills.

The challenge to adaptability and communication skills in health education will become stronger. These abilities can be used to resolve potential nurse-patient conflicts.

N4

Nursing managers further stressed that capability development should not rely solely on individual learning. Hospitals and government agencies should support training, platform development, and clear role allocation.

From a broader perspective, the government or hospitals should help departments build systems like smart nursing platforms. These systems could make information easier for nurses to use and improve efficiency.

N8

## Discussion

### Nurse-Patient Trust and Emerging Relational Tensions

This study found that AI-powered chatbots were not currently viewed by patients, caregivers, or nurses as a direct threat to nurse-patient trust. This may be because they were positioned as supplementary tools and did not replace nurses’ core professional role. However, perceptions of potential trust-related risks differed across groups, likely because of their distinct roles and responsibilities in patient education. Patients and caregivers, primarily as information seekers and users, focused more on the added informational value, convenience, and greater autonomy offered by AI-powered chatbots while still relying on nurses’ professional judgment and guidance [43]. By contrast, nurses were more concerned about patient questioning, explanatory burden, and communication pressure caused by inconsistent information. As providers of patient education and key safeguards of patient safety, they were more directly responsible for verifying information, assessing risks, and maintaining nurse-patient relationships. Previous studies similarly show that nurses recognize AI’s potential to improve efficiency and support nursing care while remaining cautious about patient safety, accountability, and changes in professional roles [44,45]. Prior research also positions AI as an adjunct and shows that patients continue to place greater trust in health care professionals [43]. However, most studies of AI and clinical trust have focused on physician-led diagnosis, treatment decisions, or clinical decision support [46,47]. This study extends the discussion to nurse-led patient education and provides new evidence on how trust may be maintained when AI enters different forms of clinical interaction.

Stakeholders differed in the conditions they considered important for maintaining nurse-patient trust after AI integration. Patients and caregivers placed greater value on nurses’ continued visible involvement and human connection. This is consistent with previous findings that patients want professional support to remain available when digital health technologies are used [43]. Such involvement can provide contextual explanations for complex or conflicting information, as well as essential safety and accountability support [48]. Nurses focused more on whether they could respond to AI-related information and provide credible explanations, suggesting that professional competence remains central to maintaining nurse-patient trust [49]. Emotional support was also emphasized across all participant groups. Care, empathy, and responsiveness remain fundamental to therapeutic relationships [49,50]. Although whether AI can offer genuine empathy remains debated, patients and caregivers felt that its continuous information support could provide some psychological reassurance when professional help was not immediately available. This aligns with uncertainty in illness theory, which suggests that adequate information support can improve understanding and reduce distress caused by uncertainty [51].

Overall, AI-powered chatbots may not change the professional support and relational interactions on which nurse-patient trust depends. Instead, they may change how these trust-maintaining mechanisms are delivered in digital settings.

On this basis, participants emphasized that patients and caregivers still required professional support from nurses when using AI-powered chatbots, consistent with previous research identifying human oversight as important for safe and trustworthy use [52]. Notably, although patients and caregivers wanted nurses’ support, some avoided discussing their chatbot use because they feared dismissal, stigma, or tension in the relationship [53]. This suggests that trust risks may arise when information from different sources conflicts but opportunities for open discussion, explanation, and integration are limited. The gap between the need for professional support and disclosure of AI use may delay the identification and resolution of inaccurate or conflicting information. AI-assisted patient education therefore requires not only continued nurse oversight and guidance but also open, nonjudgmental feedback mechanisms that support the discussion, verification, and integration of AI-generated information.

Taken together, the effects of AI-powered chatbots on nurse-patient trust should be understood from a multistakeholder perspective. Maintaining trust after their integration into patient education requires consideration of the different perspectives and needs of patients, caregivers, and nurses, together with role-appropriate strategies for each group.

### Conditional Acceptance, Digital Inequities, and Contextual Value

Patients, caregivers, and nurses were generally open but cautious about integrating AI-powered chatbots into patient education. They recognized their potential to address gaps in existing information support but remained concerned about accuracy, personalization, and transparency, consistent with previous research [24]. By providing continuous and convenient information support, AI-powered chatbots may help overcome the temporal, geographic, and resource constraints of traditional patient education, particularly where access to health care is limited, or care extends beyond hospital settings [54]. However, further functional refinement and clinical adaptation remain essential for safe implementation.

The value of AI-powered chatbots extends beyond increasing access to information. By empowering both information seekers and education providers, they may improve the quality of interactions in patient education. For patients and caregivers, chatbots can help address knowledge gaps, explain disease-related concepts, and clarify information needs, enabling a shift from passive information receipt to more prepared and active communication [55]. Caregivers may also gain the knowledge needed to participate more effectively in daily care and related decision-making. By reducing information asymmetry, chatbots may strengthen patients’ and caregivers’ ability to engage in communication and shared decision-making. Nurses primarily valued their potential to support standardized and repetitive tasks, allowing limited professional resources to be directed toward more complex and individualized education [45]. AI-powered chatbots may therefore create conditions for higher-quality patient education by supporting more informed and targeted interactions between patients, caregivers, and health care professionals.

Limited digital health literacy and the digital divide faced by older adults are important challenges to the use of AI-powered chatbots in patient education. Although chatbots reduce temporal and geographic barriers to health information, they place greater demands on users’ ability to understand, evaluate, and apply that information. People with high information needs but limited digital skills may therefore benefit less from AI, potentially widening existing inequalities in digital health [56,57]. In patient education, greater access to information does not automatically lead to knowledge gains. When digital health literacy is limited, chatbot use may instead contribute to information overload and greater illness uncertainty. Nurses may then face additional explanatory and communication demands, consistent with previous concerns that AI could increase cognitive burden and workflow pressure among health care professionals [44,58]. This highlights a digital health literacy–mediated gap between technological access and the realization of meaningful health benefits.

The suitability of AI-powered chatbots for older adults highlights both the digital divide and the need for digital empowerment. Older adults may face difficulties with device use, information seeking, and content comprehension. To support safe access to information while reducing cognitive burden, caregivers and nurses tended to favor traditional education formats with simpler operation and more intuitive content. Nevertheless, some older adults expressed a willingness to learn and use AI to manage their conditions more independently and reduce reliance on family members. Previous research similarly suggests that appropriately designed digital health tools can enhance older adults’ autonomy and sense of competence [59,60].

Accordingly, the digital transformation of patient education should not rely on the universal application of a single technological approach. Instead, traditional and AI-assisted education should be integrated within an adaptive system tailored to users’ digital health literacy, willingness to engage, and available support while promoting accessibility, equity, and patient autonomy.

### Evolving Nursing Roles and Triadic Perspectives

The use of AI-powered chatbots did not diminish nurses’ role in patient education. Instead, it broadened their responsibilities to include guiding use, verifying information, and coordinating communication. This role expansion may also create new pressures to keep knowledge current and maintain professional competence, particularly among less experienced nurses. Previous research similarly suggests that although AI can reduce routine tasks, output verification, ongoing oversight, and workflow adaptation may increase clinicians’ cognitive and practical burden [61]. Nursing capability development should therefore extend beyond basic tool use and general digital literacy. It should be aligned with nurses’ actual responsibilities in AI-assisted patient education and include AI literacy, evidence-based judgment, critical thinking, and contextual communication. Sustained training, reliable technical support, appropriate workflows, and clear accountability boundaries from health care organizations and relevant authorities are also essential [62].

Across the 3 stakeholder groups, patients’ and caregivers’ perspectives were closely aligned across most themes. This may reflect their interdependence in illness management. Although their specific responsibilities differ, both are involved in coping with and managing illness and share similar needs for information, professional support, and safety. Their assessments of the benefits and risks of AI-based information tools may therefore converge. The Theory of Dyadic Illness Management likewise conceptualizes patients and caregivers as an interdependent unit and emphasizes their mutual influence on illness perceptions, information seeking, and management behaviors [63]. Caregivers, however, placed additional emphasis on translating information into caregiving practice and supporting their participation in care-related decisions. By contrast, nurses focused more on information risks, potential tensions in nurse-patient trust, and changes in professional responsibilities arising from chatbot use. Thus, although all 3 groups shared the goal of improving patient education and protecting patients’ interests, their priorities reflected their distinct roles and responsibilities. Future studies should give greater attention in their design and analysis to stakeholders’ distinct interests and role-specific responsibilities, thereby capturing unique perspectives across groups and generating more comprehensive multistakeholder evidence.

### Implications for Safe and Equitable Integration

Feedback from patients, caregivers, and nurses indicates that safely integrating AI-powered chatbots into patient education requires coordinated action across governance, technology, user design, and clinical implementation. At the governance level, governments and professional bodies should guide the standardized development and use of health care–specific chatbots; establish regulatory and ongoing evaluation mechanisms; clarify data use, privacy protection, and accountability; and involve patients, health care professionals, and developers in system development and evaluation. These priorities are consistent with WHO (World Health Organization) [64] guidance on generative AI governance in health care.

At the technical level, high-quality clinical data and authoritative knowledge sources should be used to improve the accuracy and professionalism of AI outputs. Retrieval-augmented generation, domain adaptation, and dynamically updated patient profiles may further improve clinical relevance and personalization. User design should reduce barriers across levels of digital ability through simpler operation, readable content, multimodal interaction, and affordable access, thereby avoiding wider health inequalities [65]. Clinical implementation should proceed gradually according to task complexity, information risk, and personalization needs, with clear functional boundaries between chatbots and nurses. For highly structured, low-variability tasks, such as routine health management and common questions, chatbots may provide support under human oversight. High-risk decisions, complex explanations, and highly individualized guidance should remain led by nurses or other health care professionals. Human oversight, feedback and referral pathways, ongoing monitoring, and dynamic adjustment should also be established to ensure safety and adaptability throughout implementation [66].

### Limitations

This study has several limitations. First, the transferability of the findings may be limited by the sampling context and participant characteristics. Participants were recruited from a high-level hospital, and their educational attainment was generally high. These characteristics may limit the applicability of the findings to primary care settings and disadvantaged populations with lower educational attainment. Although purposive sampling was used to enhance sample diversity, future studies should include participants from primary care institutions, rural areas, and disadvantaged groups to further examine the applicability of the findings. Second, the interview setting may have influenced data collection. Some interviews with patients and caregivers were conducted at the bedside in shared wards. Although confidentiality was emphasized, limited privacy and the power dynamics inherent in bedside interviews within a hospital setting may have constrained participants’ willingness to express negative views. As a result, relevant dissatisfaction, criticism, or concerns may not have been fully captured. Third, participants differed in their prior experience with AI-powered chatbots. Some perspectives may have been based on limited use, and participants’ attitudes and expectations may change as AI tools mature and become more widely implemented in clinical practice.

### Conclusions

This study identified both shared and role-specific perspectives among patients, caregivers, and nurses regarding the integration of AI-powered chatbots into patient education. Participants recognized their potential as supplementary information-support tools, but acceptance remained conditional. Although chatbots were not currently perceived as a direct threat to nurse-patient trust, concerns about trust tensions and the conditions needed to maintain trust varied across stakeholder groups. Safe integration should therefore be dynamically tailored to patient characteristics, information risk, and context of use while addressing digital health literacy and the digital divide among older adults. Nurse oversight, clear accountability, and multilevel governance are essential to support a safe, equitable, and patient-centered model of human-AI collaboration.

## Appendix

Multimedia Appendix 1 COREQ (Consolidated Criteria for Reporting Qualitative Research) checklist.

Multimedia Appendix 2 Interview guide.

Multimedia Appendix 3 Characteristics of participants; research team reflexivity and credibility strategies; coding tree for themes, subthemes, and codes. Graphs are also presented showing AI-powered chatbot use by gender, educational level, and age.

## Abbreviations

COREQ: Consolidated Criteria for Reporting Qualitative Research
WHO: World Health Organization

## Data Availability

The datasets generated and analyzed in this study are available from the corresponding author (XW) upon reasonable request.
