# Supplementary material for: Integrating AI-Powered Chatbots Into Patient Education From the Perspectives of Patients, Caregivers, and Nurses: Qualitative Study
Source: J Med Internet Res. 2026 Jul 30;28:e87254. doi: 10.2196/87254 (PMC13473852; doi:10.2196/87254)
Supplement: Supplementary file 2 [file jmir_v28i1e87254_app3.docx]

Table S1. Characteristic of patients and caregivers

| Characteristics | n (%) or Mean (SD) | |
| --- | --- | --- |
| Gender, n (%) | patients (n=29) | caregivers (n=17) |
| Female | 17 (59) | 6 (35) |
| Male | 12 (41) | 11 (65) |
| Age (years), mean (SD) | 37.93 (12.73) | 37.12 (11.79) |
| ≤30 | 9 (31) | 2 (12) |
| 31～40 | 9 (31) | 9 (53) |
| 41～50 | 6 (21) | 4 (24) |
| 51～60 | 3 (10) | 2 (12) |
| ＞60 | 2 (7) | 0 (0) |
| Education level, n (%) |  |  |
| Primary school or below | 1 (3) | 0 (0) |
| Junior high | 3 (10) | 2 (12) |
| High school or Technical secondary school | 3 (10) | 7 (41) |
| Associate or bachelor’s degree | 18 (62) | 7 (41) |
| Master’s degree or above | 4 (14) | 1 (6) |
| Use of AI-powered chatbots |  |  |
| Yes | 22 (76) | 12 (71) |
| No | 7 (24) | 5 (29) |
| Residence, n (%) |  |  |
| City | 16 (55) | 7 (41) |
| Town | 7 (24) | 8 (47) |
| Village | 6 (21) | 2 (12) |
| Total monthly household income（CNY^a^）,n (%) |  |  |
| ≤5000 | 2 (7) | 1 (6) |
| 5001～10000 | 13 (45) | 10 (59) |
| 10001～20000 | 11 (38) | 3 (18) |
| ≥20000 | 3 (10) | 3 (18) |
| Length of hospital stay, n (%) |  |  |
| ≤7 | 23 (79) | 14 (82) |
| 7～28 | 4 (14) | 3 (18) |
| ≥28 | 2 (7) | 0 (0) |
| Disease type |  |  |
| Cardiovascular system | 14 (48) | 5 (29) |
| Reproductive system | 7 (24) | 2 (12) |
| Respiratory system | 5 (17) | 5 (29) |
| Musculoskeletal system | 2 (7) | 5 (29) |
| Digestive system | 1 (3) | 0 (0) |

Note. For caregivers, length of hospital stay and disease type refer to the patients for whom they provided care. ^a^CNY indicates Chinese yuan.

Table S2. Characteristics of nurses

| Nurse characteristics | n (%) or Mean (SD) |
| --- | --- |
| Gender, n (%) |  |
| Female | 14 (100) |
| Male | 0 (0) |
| Age(years), mean (SD) | 34.64 (5.85) |
| 20～30 | 5 (36) |
| 31～40 | 7 (50) |
| 41～50 | 2 (14) |
| Education level, n (%) |  |
| Bachelor’s degree | 10 (71) |
| Master’s degree or above | 4 (29) |
| Clinical Experience (years) |  |
| ≤5 | 3 (21) |
| 5～10 | 4 (29) |
| 10～20 | 6 (43) |
| ＞20 | 1 (7) |
| Department health education methods |  |
| Oral education | 14 (100) |
| Written materials | 11 (79) |
| Digital resources | 9 (64) |
| Lectures | 7 (50) |
| Patient support | 1 (7) |
| AI-powered chatbots use or not in clinical practice |  |
| Yes | 14 (100) |
| No | 0 (0) |
| Willingness to assist patients in using AI-powered chatbots |  |
| Yes | 8 (57) |
| No | 5 (36) |
| Not sure | 1 (7) |
| Department |  |
| Gynecology | 1 (7) |
| Orthopaedics | 1 (7) |
| Neurology | 1 (7) |
| Neurosurgery | 2 (14) |
| Cardiology | 5 (36) |
| Vascular Surgery | 1 (7) |
| Medical Oncology | 2 (14) |
| Intensive Care Unit (ICU) | 1 (7) |
| Position |  |
| Registered Nurse (RN) | 1 (7) |
| Nurse Practitioner (NP) | 3 (21) |
| Nurse Supervisor | 9 (64) |
| Associate Chief Nurse | 1 (7) |

Table S3. Types of Information Sought by Patients and Caregivers Using AI-Powered Chatbots (n=34)

| Type of information sought | n (%) |
| --- | --- |
| Treatment method | 20 (59) |
| Disease symptoms | 15 (44) |
| Healthy lifestyle guidance | 7 (21) |
| Disease prognosis and rehabilitation | 6 (18) |
| Medication | 6 (18) |
| Etiology | 3 (9) |
| Interpretation of diagnostic results | 3 (9) |
| Treatment costs | 1 (3) |
| Disease complication | 1 (3) |

Figure S1. AI-powered chatbot use by gender, educational level, and age group

A. AI-powered chatbot use by gender

AI-powered chatbot use by gender

Proportion, %

| Gender | Used | Not used |
| --- | --- | --- |
| Male | 73.9% | 26.1% |
| Female | 69.6% | 30.4% |

B. AI-powered chatbot use by age group

AI-powered chatbot use by age group

Proportion, %

| Age group, years | Used | Not used |
| --- | --- | --- |
| ＜30 | 69.2% | 30.8% |
| 30-44 | 73.7% | 26.3% |
| ≥45 | 71.4% | 28.6% |

C. AI-powered chatbot use by educational level

AI-powered chatbot use by educational level

Proportion, %

| Educational level | Used | Not used |
| --- | --- | --- |
| Junior high or below | 16.7% | 83.3% |
| High school/technical secondary | 70% | 30% |
| Bachelor’s/associate | 80% | 20% |
| Master’s or above | 100% | 0% |

Figure S1. AI-powered chatbot use by gender, educational level, and age group among patients and caregivers combined (n=46).

Percentages indicate the proportion of participants who reported using or not using AI-powered chatbots within each demographic subgroup.

Used = used AI-powered chatbots; Not used = did not use AI-powered chatbots.

**Researcher Reflexivity, Detailed Analytic Procedures, and Strategies for Trustworthiness**

**Researcher Characteristics and Positioning**

Interviews were conducted primarily by 2 female researchers, ZL and YL. ZL was a master’s student who had received systematic training in qualitative interviewing before the study. YL was a faculty member with a doctoral degree and training and experience in qualitative research. Her research interests included the use of AI tools in health behavior promotion and patient education. Neither interviewer was involved in participants’ diagnosis, treatment, or nursing care, and neither had a prior clinical relationship with them. Other members of the research team also had qualitative research experience and contributed different perspectives during the analysis.

The research team recognized that the interviewers’ professional backgrounds and academic interest in AI-assisted health care could influence data generation and interpretation. The team also acknowledged that power dynamics and limited privacy during bedside interviews might constrain patients’ and caregivers’ willingness to express criticism, dissatisfaction, or uncertainty.

**Preliminary Assumptions and Reflexive Considerations During Data Generation**

Before data collection, the research team discussed assumptions that might shape the study. These included expectations that AI-powered chatbots could improve access to health information and supplement existing patient education, while also introducing potential risks related to information accuracy, contextual appropriateness, patient misunderstanding, professional accountability, and nurse-patient trust.

The team also anticipated that patients, caregivers, and nurses might evaluate AI-powered chatbots differently because of their distinct roles and responsibilities in patient education. These expectations were treated as provisional assumptions to be critically examined rather than propositions to be confirmed.

During interviews, researchers used open-ended and nonjudgmental questions and actively encouraged participants to express positive, negative, conflicting, and uncertain views.

After each interview, researchers documented the interview setting, interaction process, initial impressions, personal responses, and specific ways in which the context might have influenced participants’ accounts. Reflexive notes were maintained throughout data collection and analysis and revisited during team discussions.

**Detailed Analytic Procedures**

Qualitative data were analyzed using reflexive thematic analysis following Braun and Clarke’s 6-phase framework.

(1) Data Familiarization

ZL and YL repeatedly read the full transcripts to become familiar with the data. During this phase, they recorded initial observations, questions, possible patterns, and reflexive comments. Attention was given to both shared accounts and views that appeared uncertain, contradictory, or different across participant groups.

(2) Initial Coding

The analysis was primarily inductive and semantic. Initial coding was not guided by a predefined theoretical framework or fixed coding manual, although the study objectives provided an overall direction for familiarization, coding, and interpretation. ZL conducted the initial coding of the full data set. Codes initially remained close to participants’ explicitly stated views, experiences, judgments, and concerns. Relevant extracts were coded across the data set, and analytic memos were used to document code meanings, contexts, and possible relationships. Throughout coding, the researchers reflected on how their professional backgrounds, prior assumptions, and academic interest in AI-assisted health care might shape what they noticed and how they interpreted participants’ accounts.

(3) Development of Candidate Themes

The research team compared codes to identify similarities, differences, and possible relationships. Candidate themes were developed around shared central organizing concepts rather than simply reproducing interview questions or topic summaries. To preserve differences in roles, responsibilities, and concerns, patient and caregiver accounts were initially analyzed separately from nurse accounts. Data from patients, caregivers, and nurses were then compared across groups to identify shared perspectives, role-related differences, and tensions among the 3 stakeholder groups.

(4) Reviewing and Refining Themes

Candidate themes were repeatedly reviewed against the coded extracts and the full data set. The team assessed whether each theme had a coherent central organizing concept and adequately represented participants’ accounts. Particular attention was given to negative cases, contradictory accounts, uncertain views, and differences across stakeholder groups. These accounts were not treated as analytic errors or excluded because they diverged from dominant patterns. Instead, they were used to refine theme boundaries, identify contextual variation, and avoid reducing complex experiences to a single consensus. The team also considered alternative interpretations of the data and examined whether positive views of AI-powered chatbots had been overemphasized or whether concerns related to information accuracy, nurses’ roles, and nurse-patient trust had been over- or underexplored.

(5) Defining, Naming, and Writing Themes

The research team clarified the scope and central organizing concept of each theme and its relationship to other themes. A thematic map was used to examine the overall analytic structure and relationships among candidate themes. Theme names and definitions were refined to reflect analytic interpretations rather than merely summarize interview topics. Representative quotations were selected to support the analytic claims and demonstrate the relationship between participants’ accounts and the researchers’ interpretations. Theme interpretations continued to be reviewed and refined during manuscript preparation.

**Team Involvement and Reflexive Discussion**

YL reviewed coded extracts, analytic memos, and candidate themes and engaged in ongoing reflexive discussions with ZL. Other team members read selected transcripts and reviewed candidate themes to challenge preliminary interpretations and consider alternative explanations. Through regular discussions, the team examined how researchers’ assumptions, professional backgrounds, and interest in AI-assisted health care might influence the analysis. The team also considered whether limited privacy during bedside interviews might have discouraged patients and caregivers from expressing negative views. These discussions were not intended to establish a single correct coding approach or achieve coding consensus. Instead, differences in interpretation were treated as resources for challenging assumptions, deepening understanding, and supporting the iterative development of themes. Intercoder reliability or agreement coefficients were therefore not calculated because such procedures were inconsistent with the reflexive thematic analysis approach adopted in this study.

**Trustworthiness and Analytic Transparency**

Several strategies were used to enhance trustworthiness and analytic transparency. The first author maintained sustained engagement with the full data set and documented coding decisions, candidate themes, and interpretive development through analytic memos and reflexive notes. Candidate themes were repeatedly reviewed against both coded extracts and the broader data set. Particular attention was given to negative cases, divergent views, uncertain accounts, and differences across stakeholder groups. Researchers who were not responsible for the primary coding also participated in critical discussions and considered alternative interpretations. NVivo coding records, analytic memos, reflexive notes, and records of theme revisions were retained to document the progression from raw transcripts to the final themes. Participant quotations were included in the findings to allow readers to examine the relationship between participants’ accounts and the analytic interpretations.

**Rationale for Not Conducting Member Checking**

Transcripts were not returned to participants for correction, and participants were not invited to comment on preliminary themes. This decision reflected patients’ and caregivers’ illness or caregiving burden, the limited feasibility of maintaining contact after discharge, and the potential additional time burden for nurses. As alternatives to member checking, trustworthiness was supported through sustained engagement with the full data set, reflexive documentation, repeated review of themes against the data, attention to negative and discrepant accounts, cross-group comparison, and critical discussion among researchers with different perspectives.

**Coding Tree for Themes, Subthemes, and Codes**

Patient, caregiver, and nurse accounts were initially examined separately to preserve the distinct perspectives associated with each participant group. During theme development, patterns of meaning were compared across the three groups and brought together within a single overarching thematic framework that incorporated both shared and role-specific perspectives. Items derived primarily from nurse accounts are marked as (nurse) in the coding tree.

Table S4. Coding Tree for Themes, Subthemes, and Codes

| Theme | Subtheme | Codes / Nodes |
| --- | --- | --- |
| Perceptions and Maintenance of Nurse-Patient Trust in the Use of AI-Powered Chatbots for Patient Education | Supplementary Positioning and Emerging Trust Tensions | Auxiliary positioning of AI-powered chatbots |
|  |  | Trust in health care professionals remains dominant |
|  |  | Potential trust pressure caused by inconsistent information (nurse) |
|  | Conditions for Maintaining Nurse-Patient Trust | Emotional support provided by nurses |
|  |  | Fit with nurses’ professional competence (nurse) |
|  |  | Patients’ cognitive level |
| Attitudes Toward Integrating AI-Powered Chatbots Into Patient Education and Real-World Considerations | Conditional Support | Conditional support |
|  |  | Cautious attitude |
|  |  | Contextual dependence (nurse) |
|  | Major Concerns | Insufficient health literacy and information appraisal ability |
|  |  | Increased educational burden for nurses (nurse) |
|  |  | Age constraint |
|  |  | Technical limitations (accuracy and personalization) |
|  |  | Insufficient system support (nurse) |
|  | Real-World Foundations for Use | Limited workforce and time resources |
|  |  | Insufficient primary care resources |
|  |  | Optimize nursing time allocation (nurse) |
|  |  | Insufficient continuity of postdischarge education support |
| Optimization Needs and Implementation Safeguards for Integrating AI-Powered Chatbots Into Patient Education | Functional Optimization Needs | Improving accuracy and personalization |
|  |  | Establishing a medical-specific platform |
|  | Professional Oversight and Open Feedback | Nurse supervision and guidance as prerequisites for implementation |
| Nurses’ Role Pressure and Capability Reconfiguration in Patient Education | Role Pressure | Competence uncertainty |
|  |  | Pressure to update knowledge |
|  | Capability Reconfiguration and the Need for Organizational Support | Improving professional skills |
|  |  | Need for professional knowledge updating |
|  |  | Improving ability to use AI tools |
|  |  | Need to improve critical thinking and evidence-based competence |
|  |  | Improving communication skills |
|  |  | Strengthening psychological resilience |
|  |  | Need for organizational and policy support |

Table S5. Shared and Role-Specific Perspectives of Patients, Caregivers, and Nurses on Integrating AI-Powered Chatbots Into Patient Education

| Theme | Subtheme | Caregiver Perspective | Patient Perspective | Nurse Perspective | Shared Views and Role-Specific Emphases |
| --- | --- | --- | --- | --- | --- |
| Theme 1: Perceptions and Maintenance of Nurse-Patient Trust in the Use of AI-Powered Chatbots for Patient Education | Subtheme 1: Supplementary Positioning and Emerging Trust Tensions | Viewed AI-powered chatbots as supplementary information sources, mainly for obtaining disease-related information and supporting understanding; continued to rely heavily on health care professionals’ judgment. | Viewed AI-powered chatbots as supplementary information sources, mainly for obtaining disease-related information and supporting understanding; continued to rely heavily on health care professionals’ judgment. | Mainly used AI-powered chatbots for basic supportive tasks; concerned that inconsistent information could lead to patient questioning and communication pressure. | All three groups recognized AI’s supplementary role. Patients and caregivers emphasized its value in supplementing information and continued to trust health care professionals, whereas nurses were more sensitive to potential trust tensions arising from inconsistent information. |
|  | Subtheme 2: Conditions for Maintaining Nurse-Patient Trust | Emphasized nurses' continued involvement, emotional support, and communication quality; also considered AI system maturity and users’ ability to understand the information. | Emphasized nurses’ continued involvement, emotional support, and communication quality; also considered AI system maturity and users’ ability to understand the information. | Emphasized emotional support, professional competence, users’ cognitive ability, and AI system maturity. | Emotional support, communication quality, AI system maturity, and users’ cognitive ability were conditions emphasized by all three groups. Patients and caregivers placed greater value on nurses’ continued involvement and human connection, whereas nurses focused more on their own professional competence. |
| Theme 2: Attitudes Toward Integrating AI-Powered Chatbots Into Patient Education and Real-World Considerations | Subtheme 1: Conditional Support | Recognized the comprehensiveness and convenience of information provided by AI-powered chatbots but were concerned about insufficient accuracy, personalization, and functional maturity. | Recognized the comprehensiveness and convenience of information provided by AI-powered chatbots while also considering accuracy, personalization, source transparency, and functional maturity. | Recognized the comprehensiveness and convenience of information provided by AI-powered chatbots while also considering accuracy, personalization, source transparency, functional maturity, and contextual dependence. | All three groups held a cautious yet open attitude and shared concerns about insufficient accuracy and personalization and the level of functional maturity. Patients and nurses also considered source transparency, whereas nurses placed greater emphasis on contextual dependence. |
|  | Subtheme 2: Major Concerns | Were concerned about limited medical knowledge and ability to evaluate information, as well as the digital divide among older patients. | Were concerned about limited medical knowledge and ability to evaluate information, as well as the digital divide among older patients; some older patients expressed a willingness to learn proactively. | Focused on limited digital health literacy among patients and caregivers, the digital divide among older adults, and the additional explanatory burden caused by information misunderstanding. | All three groups were concerned about limited digital health literacy and the digital divide among older adults. Nurses were concerned about increased explanatory burden, whereas some older patients demonstrated a need for digital empowerment. |
|  | Subtheme 3: Real-World Foundations for Use | Limited time for inpatient education; insufficient out-of-hospital follow-up. | Limited primary care resources; limited time for inpatient education; insufficient out-of-hospital follow-up. | Limited primary care resources; limited time for inpatient education; insufficient out-of-hospital follow-up. | All three groups considered the time available for existing patient education and out-of-hospital follow-up insufficient. Patients and nurses emphasized limited primary care resources. |
| Theme three: Optimization Needs and Implementation Safeguards for Integrating AI-Powered Chatbots Into Patient Education | Subtheme 1: Functional Optimization Needs | Emphasized improving accuracy and personalization while also considering privacy and security, accessibility, affordability, and gradual implementation. | Emphasized improving accuracy, personalization, privacy and security, accessibility, and affordability, and supported phased implementation. | Emphasized improving accuracy and personalization and strengthening supporting conditions. | Improving accuracy and personalization was a shared priority across all three groups. Patients and caregivers placed greater emphasis on safety, accessibility, and affordability, whereas nurses focused more on improving supporting conditions. |
|  | Subtheme 2: Professional Oversight and Open Feedback | Wanted nurses to verify accuracy and guide appropriate use but avoided proactively disclosing information obtained through AI searches. | Wanted nurses to verify accuracy and guide appropriate use but avoided proactively disclosing information obtained through AI searches. | Emphasized professional review of AI-generated information and guiding patients and caregivers to formulate more accurate questions and use the tools appropriately. | All three groups recognized the need for nurse oversight and guidance. Patients and caregivers tended to avoid discussing their AI use. |
| Theme 4: Nurses’ Role Pressure and Capability Reconfiguration in Patient Education | Subtheme 1: Role Pressure |  |  | Pressure to update knowledge and challenges to perceived professional competence. |  |
|  | Subtheme 2: Capability Reconfiguration and the Need for Organizational Support |  |  | A need to continually update professional knowledge and strengthen AI literacy, critical thinking, evidence-based judgment, and contextual communication skills, together with organizational support. |  |
