# Supplementary material for: Integrating AI-Powered Chatbots Into Patient Education From the Perspectives of Patients, Caregivers, and Nurses: Qualitative Study
Source: J Med Internet Res. 2026 Jul 30;28:e87254. doi: 10.2196/87254 (PMC13473852; doi:10.2196/87254)
Supplement: Supplementary file 3 [file jmir_v28i1e87254_app2.doc]

**Semi-structured interview guide**

**Patients and Caregivers**

Patients and caregivers were interviewed using the same core questions because both groups were asked about similar domains related to information seeking, AI-powered chatbot use, patient education, and implementation needs. Interviewers adapted the wording and follow-up probes to the participant’s role. Patients described their own experiences, whereas caregivers described their experiences of seeking, evaluating, and using information while supporting the patient. Caregivers were interviewed as independent participants rather than as proxies for patients.

1. Where do you usually obtain disease-related information for yourself or the patient you care for?
2. Are you familiar with AI-powered chatbots ?
   2.1. Yes
   2.2. No → Would you like to learn how to use AI-powered chatbots?
        2.2.1. Yes → Would you be willing to use an AI-powered chatbot to look up disease-related information? Why or why not?
        2.2.2. No →Why not?
3. Do you used AI-powered chatbots (e.g., Deepseek, Doubao) to look up disease-related information for yourself or the patient you care for?
   3.1. No → Please describe the reasons

Follow up: What concerns or worries do you have about using AI-powered chatbots?

3.2. Yes → What topics do you usually search for?

(e.g., etiology, symptoms, treatment options, prognosis, medication, drug functions, etc.)

Follow up: ①How do you generally view the results provided by AI-powered chatbots?

②What motivates you to use the chatbot?

③Compared with nurses’ education, which do you trust more: information from AI-powered chatbots or from healthcare professionals? Why?

④What concerns or worries do you have about using AI-powered chatbots?

4. Has the information you obtained from AI-powered chatbots ever conflicted with the patient education provided by nurses?
   4.1. If Yes → How do you view the conflicts ? How do you handle the conflicts?

Follow up: When conflicts occur, do you consult a nurse? What kinds of clarification or guidance do nurses usually provide?

5. Would you support integrating AI-powered chatbots into clinical patient education?
   5.1. No →Why not?
   5.2. Yes → What suggestions or expectations do you have for introducing AI-powered chatbots into clinical patient education?

Follow up: When using AI-powered chatbots to search for disease information, what kinds of support would you like to receive from healthcare staff?

**Nurses**

1. In your daily work, how do you provide patient/ caregiver education?

□One-to-one verbal education □ Printed materials □ Media resources (e.g., WeChat official account, Unit QR codes) □ Classroom/ lecture-style teaching □ Other:

2. Are you familiar with AI-powered chatbots?
3. In your work, have you encountered patients/ caregivers who use AI-powered chatbots to look up disease-related information?
   3.1. If no → Would you be willing to accept/ approve patients/ caregivers using AI-powered chatbots to search for health information? Why or why not?
   3.2. If yes → What is your overall view of patients/ caregivers using AI-powered chatbots?
4. Have you encountered situations where AI-powered chatbots information conflicted with your education content?

If conflicts occurred →① How did you feel at the time?

②Do you think such situations could create a nurse-patient trust crisis?

③What strategies did you use (or would you use) to resolve the conflict?

5. Would you be willing to integrate AI-powered chatbots into patient education?
   5.1. If no → Why not?
   5.2. If yes → In your view, how could AI-powered chatbots be best applied to your work, especially in patient education?
6. Would you be willing to help patients/ caregivers use AI-powered chatbots for health management more effectively?
   6.1. If no → Why not?
   6.2. If yes → What specific measure would you take?
7. What challenges do you think nurses will face when AI-powered chatbots are used in patient education?

Follow up: What preparations should nurses make, or what competencies should be strengthened?
